# Analysis of the Correlation between Meal Frequency and Obesity among Chinese Adults Aged 18–59 Years in 2015

**DOI:** 10.3390/nu14030696

**Published:** 2022-02-07

**Authors:** Xiaoqi Wei, Dongmei Yu, Lahong Ju, Qiya Guo, Hongyun Fang, Liyun Zhao

**Affiliations:** Key Laboratory of Trace Element Nutrition of National Health Commission, National Institute for Nutrition and Health, Chinese Center for Disease Control and Prevention, Beijing 100050, China; xq437073568@163.com (X.W.); yudm@ninh.chinacdc.cn (D.Y.); julh@ninh.chinacdc.cn (L.J.); guoqy@ninh.chinacdc.cn (Q.G.); fanghy@ninh.chinacdc.cn (H.F.)

**Keywords:** meal frequency, obesity, two-level model, eat away from home, eating at dining halls

## Abstract

This study aimed to investigate the relationship between meal frequency and obesity in Chinese adults aged 18 to 59 years. The data came from the 2015 Chinese Adult Chronic Disease and Nutrition Surveillance (CACDNS 2015) and provincial dietary environment data from the 2015 National Statistical Yearbook. A total of 34,206 adults aged 18 to 59 who took part in the diet survey were selected as the study participants. A two-level multivariate logistic regression model was used to adjust for the socioeconomic and nutritional status of individuals. For parameter estimation, a numerical integral approach was used to analyze the relationship between meal frequency (including meals at home, the workplace or school dining halls, and eating away from home) and obesity. A two-level “provincial–individual” logistic multivariate regression analysis was performed with obesity as the dependent variable. The two-level multivariate analysis of variance model fitting results showed that after adjusting for the effects of gender, age, occupation, education, marital status, family per capita annual income, provincial gross domestic product (GDP), restaurant industry turnover, consumer price index of EAFH food, and energy intake, the frequency of eating at home was not associated with obesity (all *p* > 0.05); the frequency of eating at dining halls ≥1 to <2 times per day (OR = 0.784, *p* = 0.0122) showed a negative association with obesity; the frequency of eating away from home < 1 times per day and ≥1 to <2 times per day were positively correlated with obesity (<1 time per day: OR = 1.123, *p* = 0.0419; ≥1 to <2 times per day: OR = 1.249, *p* = 0.0022). The results of the two-level random-intercept logistic multivariate mixed-effects prediction model for obesity in adults aged 18 to 59 years showed that no statistical association was noticed between the frequency of eating at home and obesity in adults aged 18 to 59 years. However, adults who ate out < 1 time and ≥1 to <2 times a day showed higher risks of obesity than those who did not eat out, with OR = 1.131 (95% CI 1.012–1.264) and OR = 1.258 (95% CI 1.099–1.440), while adults who ate at school and workplace dining halls ≥1 to <2 times a day may have a reduced risk of obesity, with OR = 0.790 (95% CI 0.656–0.951). This result could not be found based on the definition of eating out in previous studies. Therefore, it is recommended to exclude nonprofit collective canteens such as school and workplace dining halls from the definition of eating away from home, and to redefine eating out in terms of health effects. At the same time, it is also recommended to strengthen collective nutritional interventions around canteens, improve the nutritious meal system in school and workplace canteens, and create healthy canteens.

## 1. Introduction

According to the National Bureau of Statistics, China’s gross domestic product (GDP) exceeded $15 trillion ($14.725 trillion) for the first time in 2020. As China’s economy has diversified and consumers have become more focused on convenience and speed, the catering industry and takeaway market have experienced explosive growth in recent years [1]. From 2015 to 2017, the proportion of residents aged 6 years and above who ate away from home (EAFH) in China reached 46.3%, with 33.2% of adults aged 18 to 44 years and 19.8% of adults aged 45 to 59 years [2]. Especially during the COVID-19 epidemic, many branded catering industries and food markets have adopted contactless distribution methods to eliminate the risks of uncertainty associated with potential safety hazards and infection fears by diversifying sales channels by increasing takeout options and providing consumers with better services and maximum convenience [3]. The proportion of eating out not only in China but all over the world has shown a substantial increase. According to the NHANES, the proportion of American adults eating out from 2005 to 2014 was about 34.0%. The percentage of people who ate out at least once a day was as high as 64% [4,5]. From 2008 to 2012, the percentage of adults aged 19 and over eating out in the UK was 27.1%, of which 21.1% ate takeaway at least once a week [6]. The percentage of Koreans aged 19 years and older who ate out over 1 time a day increased from 24.7% in 2008 to 31.0% in 2019 [7]. The percentage of Japanese adults aged 20 and above eating out at least 1 time a week increased from 32.3% in 2015 to 33.6% in 2019 [8].

Studies at home and abroad have found the relationship between the frequency of EAFH and obesity. Bhutani’s study found that for every additional meal at fast-food and sit-down restaurants, BMI increased by 0.8 kg/m^2^ and 0.6 kg/m, respectively [9]. Kim’s study found that EAFH more than 7 times a week increased the risk of BMI and obesity [10]. In the 2015 Chinese Nutrition and Health Survey (CNHS 2015), the frequency of eating out for 18–59-year-old males was 14–21 times per week, and the risk of obesity was 1.8 times that of the non-eating-out group [11]. With economic development and social progress, EAFH will become an indispensable part of life. Studies have shown that students and on-the-job personnel are the dominant groups of people who eat out. In Shanghai, China, 70% of people prefer to have lunch in the company and school canteens [12]. Studies have found that school and workplace canteens can provide nutritionally balanced food, and school and workplace food services can contribute to healthy eating habits [13,14]. In order to implement the “Healthy China 2030” Planning Outline, the National Health Commission of the People’s Republic of China has planned the “Guidelines for the Construction of Nutritious and Healthy Canteens” to guide the catering industry to enhance the awareness of nutrition and health, and encourage canteens to provide special nutritional meals to people with obesity or nutrition-related diseases, to create a nutritious and healthy dining atmosphere [15]. However, in most of the current studies on EAFH, school and work canteens are included in the definition of EAFH, which confounds the analysis of real eating out and health-related outcomes [16]. Therefore, this study analyzed the association between meal frequency (including meals at home, workplace or school dining halls, and eating away from home) and obesity among adults aged 18–59 by using the dietary data from the 2015 China Adults Chronic Diseases and Nutrition Surveillance (CACDNS 2015), which was a major public health project of the Former National Health Commission of the People’s Republic of China and the Chinese Center for Disease Control and Prevention, to provide the basis for reformulating the definition of EAFH and obese risk control strategy.

## 2. Materials and Methods

### 2.1. Study Design and Samples

The data were derived from the 2015 China Adults Chronic Diseases and Nutrition Surveillance (CACDNS 2015). A multi-stage stratified cluster random sampling method was used to sample the population of 31 provinces, autonomous regions, and municipalities [11,16]. After data cleaning, 34,206 adults aged 18 to 59 years were included in this study. This project was approved by the Ethics Review Committee of the Chinese Center for Disease Control and Prevention (No. 201519-B), and all respondents signed informed consent before participation. In addition, the provincial dietary environment data [1] from the 2015 National Statistical Yearbook were used to analyze the effect of provincial variables on obesity, mainly including China’s provincial gross domestic product (GDP), restaurant turnover, food consumption price index for food eaten out, and per capita daily consumption of edible oil and vegetables.

### 2.2. Data Collection and Measurements

The CACDNS 2015 conducted the dietary survey and physical measurement among the respondents through an inquiry survey [11]. The dietary survey was conducted by face-to-face interviews, including a weighted dietary record, 3-day 24 h dietary recalls, and a food frequency questionnaire (FFQ) survey. Household consumption of edible oil and major condiments for 3 consecutive days (two weekdays and one weekend day) was investigated by a weighing dietary record method. Three-day 24 h dietary recalls were used to collect all the food that the respondents ate at home and out for 24 h for 3 consecutive days (two weekdays and one weekend day), including the staple food, non-staple food, snacks, fruits, and drinks. FFQ was used to ask respondents the frequency at which they consumed given foods and the amount of each consumption in the past 12 months or 1 month. The physical examination included indicators such as height and weight. Height and weight were measured centrally by a uniformly trained investigator using a certified measurement instrument (TZG type height and sitting altimeter and Tanita HD-390 electronic weight scale) designated by the National Project Team and approved by metrology certification. The readings were accurate to 0.1 cm and 0.1 kg, respectively. When measuring height and weight, subjects were required to take off their shoes and hats, and wear lightweight clothes.

### 2.3. Criteria of Obesity

Body mass index (BMI) was calculated with the following formula: body weight (kg)/height^2^ (m^2^). According to the Criteria of Weight for Adults of the health industry standard of China, WS/T 428–2013 [17], the categories of BMI are as follows: underweight, BMI <18.5 kg/m^2^; normal, 18.5 kg/m^2^ ≤ BMI < 24 kg/m^2^; overweight, 24 kg/m^2^ ≤ BMI < 28 kg/m^2^; and obesity, BMI ≥ 28 kg/m^2^.

### 2.4. Covariates

The variables at the individual level were age, sex, marital status, occupation, education level, frequency of meals eaten at home, dining halls, and away from home, energy intake, fat energy ratio, sodium intake, and household per capita annual income. The variables at the provincial level were provincial gross domestic product, catering industry turnover, food consumption price index for food eaten out, and per capita daily consumption of edible oil and vegetables [1,18].

### 2.5. Definition of Relevant Independent Variables

(1) “Frequency of EAFH” was defined as the respondents eating away from home at least once in the past 7 days or eating food not prepared at home as a main meal at home. Dining places were divided into five types: buy foods and eat them at home (takeaway, ordering, and box meals), Chinese restaurant (including Chinese fast-food restaurant), Western restaurant (including western fast-food restaurant), bakery or cake shops or coffee shops, and other places (excluding eating at workplace and school dining halls). (2) “Frequency of Home” was defined as the frequency of eating home-made or prepared food at home in the past 7 days. (3) “Frequency of EADH” was defined as the frequency of eating at the workplace and school dining halls in the past 7 days. (4) Provincial gross domestic product (GDP, 100 million yuan) refers to the final output of production activities of all resident units in each province within a certain period of time calculated at market prices. (5) Catering industry turnover (100 million yuan) refers to the income obtained by the catering business unit from providing services or selling goods in its business activities, including income from various foods sold after cooking, preparation and processing, such as staple foods, stir-fried vegetables, and cold dishes. (6) Food consumption price index for food eaten out refers to the relative value of the price change trend and the degree of food (including staple foods, stir-fried vegetables, local snacks, and Chinese and Western fast food) that is eaten in the catering industry during meals outside the home in urban and rural areas within a certain period. (7) Per capita daily consumption of edible oil (kg) refers to edible oils and fats consumed by urban and rural households in a year, including various vegetable oils extracted from plant fruits, such as olive, corn, soybean, and peanut oil, and various raw and cooked animal oils, such as lard, as well as butter, butter products, margarine, and other vegetable fats. (8) Per capita daily consumption of vegetables (kg) refers to the quantity of vegetables consumed by urban and rural households in a year, including fresh vegetables, dried vegetables, fresh fungi, dried fungi, vegetable products, and mushroom products [1].

### 2.6. Statistical Analysis

SAS software (version 9.4, SAS Institute Inc., Cary, NC, USA) was used for data cleaning and analysis. The intake data of the average daily energy, sodium, and fat energy ratio were centrally processed according to the recommended values for different genders and age groups in “Dietary Nutrient Reference Intake of Chinese Residents (2014 edition)” [19] issued by the Chinese Nutrition Society, and median values were centrally processed for other continuous variables. In this study, a two-level logistic regression model was used to analyze the influencing factors. First, the PROC GLIMMIX procedure was used to obtain the estimated value of the parameters, and then the PROC NLMIXED procedure was used to obtain the results of the two-level logistic regression model and the goodness-of-fit test. Parameter estimation was conducted using *t*-test numerical integral approximation; *p* < 0.05 was considered statistically significant. The basic form of the two-level logistic regression model adopted in this study is

$$Logit\left(P_{ij}\right)=\beta_{0}+\beta_{1x1_{ij}}+\ldots +\beta_{nxn_{ij}}+\mu_{0j}$$

$\mu_{0j}$~N (0, $\sigma_{u_{0}}^{2}$), $\mu_{0j}$ represents the difference between the Logit mean ($\beta_{0j}$) at level 2 (provincial units) and the population mean ($\beta_{0}$), that is, the random effect at level 2 (provincial units), and its variance is $\sigma_{u_{0}}^{2}$, which is used to represent the difference in obesity rate among provincial units, and OR = exp (*β*).

## 3. Results

### 3.1. Participant Characteristics

Table 1 shows the basic characteristics of the surveyed subjects. A total of 34,206 respondents were surveyed in 31 provinces (autonomous regions and municipalities), including 15,093 males, accounting for 44.12%, and 19,113 females, accounting for 55.88%; 14,850 people aged 18 to 44, accounting for 43.41%, and 19,356 people aged 45 to 59, accounting for 56.59%. A total of 93.26% were married or cohabiting, 35.53% were on-the-job personnel (excluding agriculture, forestry, animal husbandry, fishing, and water conservation), 10.09% had a junior college education and above, and 14.62% were obese. The data distribution of continuous variables is shown in Table 2. The population distribution of 31 provinces (autonomous regions and municipalities) is shown in Table A1.

### 3.2. Multilevel Logistic Model Regression Results

#### 3.2.1. Fitting Results of the Empty Model

In CACDNS 2015, 302 surveillance sites were randomly selected from each province (autonomous regions and municipalities), 3 towns (streets) were randomly selected from each monitoring site, and 2 villages (neighborhood committees) were randomly selected from each town (street). The affiliation makes the data aggregated in the region. Taking obesity (1 = yes, 0 = no) as the dependent variable, a random effect test of the null model with two levels of “provincial–individual” was carried out. The result in Table 3 was statistically significant at *p* = 0.05, and the data had aggregation at level 2. Therefore, this data was suitable for a two-level logistic model analysis.

#### 3.2.2. Fitting Results of the Two-Level Multi-Factor Logistic Model

Table 4 shows the results of the fitted two-level multivariate analysis of variance model after introducing all the independent variables. After adjusting for gender, age, marital level, employment, education level, per capita annual household income, provincial GDP, catering industry turnover, food consumption price index for eating out, and energy intake, the frequency of eating at home was not associated with obesity (all *p* > 0.05). The frequency of EADH ≥1 to <2 times per day (OR = 0.784, *p* = 0.0122) was negatively correlated with obesity. The frequency of EAFH <1 time and ≥1 to <2 times per day group was positively correlated with obesity (<1 time per day: OR = 1.123, *p* = 0.0419; ≥1 to <2 times per day: OR = 1.249, *p* = 0.0022).

#### 3.2.3. Fitting Results of the Optimized Two-Level Multi-Factor Logistic Prediction Model

A two-level logistic multivariate random intercept mixed effect prediction model for obesity in adults aged 18–59 was obtained by fitting the statistically significant factors. The model fitting results are shown in Table 5. The main meal frequency factors affecting obesity were the frequency of EAFH and EADH. After adjusting for other factors, it can be seen that the frequency of EADH is a protective factor for obesity (OR = 0.790, t = −2.59, *p* = 0.0145). The frequency of EAFH is a risk factor for obesity. Adults who EAFH < 1 time per day had about a 1.131 times higher risk of obesity than those who did not EAFH, and adults who EAFH ≥1 to <2 times per day had a 1.258 times higher risk of obesity than those who did not EAFH. However, the frequency of eating at home and the frequency of EADH <1 time and ≥2 times a day were not associated with obesity. Model fitting information is shown in Table A2.

## 4. Discussion

The relationship between dietary frequency and obesity was analyzed by fitting an optimized two-level multivariate logistic prediction model method. The results showed that the frequency of EAFH (excluding school and workplace dining halls) was a risk factor for obesity, while the frequency of eating at school and workplace dining halls was a protective factor, and the frequency of eating at home was not associated with obesity.

Over the past 40 years, global obesity rates have risen from less than 1% in 1975 to 6–8% in 2016, with male obesity rates increasing from 3% to 11% and female obesity rates increasing from 6% to 15% over the same period [20]. In China, the obesity rate rose from 3.1% in 2010 to 8.1% in 2018 [21]. Besides lack of physical activity, one of the major causes of obesity is a change in eating habits, where the energy obtained from food exceeds the body consumption, leading to the accumulation of body fat [22]. EAFH food consumption data showed that people eat more food while EAFH, and EAFH foods were energy-dense, bulky, and nutrient-dense. The dietary energy intake and dietary energy density (food energy/food weight) of restaurant diners are significantly higher than those of diners at home [23,24]. In the United States, for example, the proportion of energy provided by EAFH for children aged 2–18 increased from 23.4% in 1977 to 33.9% in 2006, with fast food accounting for more energy than school meals [25]. From 2003 to 2010, the total daily energy of fast-food and full-service restaurants for adults aged 18 and older in the United States increased by 190.29 kcal and 186.74 kcal, respectively [23]. The British Nutrition Survey shows that adults who EAFH at least 1 time a week consume an average of 75–104 kcal more per day than those who do not EAFH, and those who eat takeaway at home consume 63–87 kcal more per day than those who do not eat takeaway [26]. Therefore, regular or long-term EAFH may lead to higher energy intake and higher body fat, which may increase the risk of obesity [27].

Where food comes from and where it is consumed are both related to the quality of the diet [28]. The definition of EAFH in current domestic and foreign studies usually refers to eating at all places except home including Chinese and Western restaurants, Chinese and Western fast-food restaurants, cafeterias, school and workplace dining halls, convenience stores, and bakeries and stalls [2,29]. However, the definition of EAFH in this study excludes school and workplace dining halls. Meanwhile, this study also found that the frequency of EAFH excluding school and workplace dining halls is negatively correlated with obesity. Adults who EAFH <1 time per day had about a 1.131 times higher risk of obesity than those who did not EAFH, and adults who EAFH ≥1 to <2 times per day had a 1.258 times higher risk of obesity than those who did not EAFH. Therefore, it can be inferred that the risk of obesity is on the rise with the increasing frequency of EAFH. Judy Kruger ’s study found that adults who did not eat at fast-food restaurants were more successful at maintaining weight loss than those who ate at fast-food restaurants two or more times a week, meaning that eating less often at fast-food restaurants helped maintain weight loss [30]. Ma’s study found that participants who frequently EAFH had an approximately two-fold increased risk of obesity compared to those who rarely EAFH [31]. A study in China found that men who EAFH 3 times or more per week were 1.53 times more likely to be obese than those who had not EAFH in the past month. For women, the risk of being obese was 2.23 times higher than those who did not EAFH in the past month [32]. Therefore, the frequency of EAFH excluding non-profit school and workplace canteens is one of the risk factors for obesity.

In this study, the frequency of eating at home was not associated with obesity. In multiple studies, food prepared and eaten at home has been associated with higher quality diets and better health outcomes. Tiwari’s study found that frequent home cooking was associated with a lower energy intake and reduced sugar and fat intake [33]. In addition, Susanna Mills’ study found that frequent home cooking was associated with higher fruit and vegetable intake and higher vitamin C levels. People who ate at home over five times per week can eat 62.3 g more of fruit and 97.8 g more of vegetables per day. People who ate at home over five times per week were 28 percent less likely to be overweight than those who ate at home less than three times a week [34]. Therefore, frequent cooking at home may be better for diet and health. The study also found that the frequency of school and workplace dining halls was a protective factor for adult obesity. After adjusting for individual social factors such as gender, age, marital level, employment, education level, per capita annual household income, and provincial economic factors such as GDP, the frequency of EADH ≥1 to <2 times per day is a protective factor for obesity. From 2015 to 2017, the percentage of Chinese adults aged 18 to 44 who ate breakfast, lunch, and dinner at school or workplace canteens was 3.4, 11.4, and 2.9%, respectively [2]. Most schools in China are equipped with canteens, and students usually eat at school canteens. The food chosen by the school canteen is a key factor affecting the improvement effect of students’ nutrition. In July 2013, the Health Life Style Action State Office issued “Health life style action supporting environment construction guidelines”, which specifies the definition of a “healthy canteen” and evaluation criteria; evaluation contents include the four aspects of basic conditions, personnel requirements, the dining room environment, and meals served [35]. School and workplace canteens provide centralized dining places for students and workers, and their main purpose may not be to make profits, but to meet the need of students and workers for nutrition and health [36]. In China, the proportion of people who ate lunch at the company or school canteens was about 70%, and canteen set meals are the most popular source of lunch for urban office workers [12,37]. Studies have found that school and workplace canteens can provide nutritionally balanced food, and that school and workplace dietary services can help foster healthy eating habits [13,14]. Lassen’s intervention study [38] showed that dietary patterns in group canteens such as workplace and school canteens may be more reasonable than in restaurants or takeaways, with the amount and variety of food served playing a crucial role in the food consumption choices of many office workers at lunchtime. Roos’ study [13] also showed that eating lunch at workplace canteens was associated with a lower BMI. It may be related to the balanced food provided by the workplace canteen for employees to choose from and employees eating more vegetables at lunch, which was consistent with the research results of Campbell’s study [39]. Therefore, the analysis of EAFH frequency and health-related outcomes using the current definitions of EAFH in domestic and foreign studies is mixed to some extent, and collective canteens such as school or workplace canteens should be separated from for-profit dining places such as restaurants. In situations where rational dietary patterns in collective canteens such as school and workplace canteens are confused, the health effects of irrational dietary patterns in restaurants and other for-profit dining places are masked and underestimated. Therefore, it is recommended to exclude non-profit collective dining halls such as school and workplace canteens from the definition of eating away from home and redefine eating away from home from the perspective of affecting health outcomes. In addition, the spread of COVID-19 may affect the catering industry for a long time. Therefore, consumers should reduce the opportunities to eat in public places, and are encouraged to eat at less crowded places, such as private restaurants, school or workplaces canteens, or at home [40,41].

In this study, by fitting a provincial and individual two-level multivariate logistic model, it is concluded that the frequency of eating at home was not associated with obesity, while the frequency of EAFH <1 time and ≥1 to <2 times per week was a risk factor for obesity, and the frequency EADH ≥1 to <2 times per week was a protective factor for obesity. However, this study still has some limitations. First, the data in this study are from a cross-sectional study, which cannot explain the causal relationship between meal frequency and obesity. Second, this study did not consider the effect of physical activity on obesity. Therefore, future studies will be considered in combination with physical activity levels and additional prospective studies.

## 5. Conclusions

In conclusion, the frequency of eating at home was not associated with obesity, while the frequency of EAFH <1 time and ≥1 to <2 times per week was a risk factor for obesity, and the frequency EADH ≥1 to <2 times per week was a protective factor for obesity. This result could not be found based on the definition of eating away from home used in previous studies. Therefore, it is recommended to exclude non-profit collective dining halls such as school and workplace canteens from the definition of EAFH and redefine EAFH from the perspective of affecting health outcomes. At the same time, it is suggested to strengthen the canteen at the center of collective canteen meal intervention work and improve school and workplace canteens’ nutritious meal system to create healthy canteens.

## Figures and Tables

**Table 1:** Data distribution and assignment of classification variables.

Variable			*N*	%	Assignment
Dependent variable	Obesity	No (ref)	29,204	85.38	0
		Yes	5002	14.62	1
Level 1—individual	Sex	Male (ref)	15,093	44.12	1
		Female	19,113	55.88	2
	Age, years	18~44 (ref)	14,850	43.41	1
		45~59	19,356	56.59	2
	Marital Level	Spinsterhood (ref)	1692	4.95	1
		Married/cohabitation	31,900	93.26	2
		Widowed/divorce/separation	614	1.80	3
	Employment	Farming and aquaculture (ref)	15,115	44.19	1
		Others	12,152	35.53	2
		Student	142	0.42	3
		Unemployed/retired	6797	19.87	4
	Education Level	Junior high and below (ref)	25,210	73.70	1
		High school/technical secondary school/technical school	5543	16.20	2
		Junior college and above	3453	10.09	3
	Household Income Level, yuan	<10,000 (ref)	14,209	41.54	1
		10,000~19,999	10,879	31.80	2
		≥20,000	9118	26.66	3
	Frequency of Home, per day	No (ref)	356	1.04	1
		<1 time	793	2.32	2
		≥1 to <2 times	3025	8.84	3
		≥2 times	30,032	87.80	4
	Frequency of EADH, per day	No (ref)	31,126	91.00	1
		<1 time	1163	3.40	2
		≥1 to <2 times	1488	4.35	3
		≥2 times	429	1.25	4
	Frequency of EAFH, per day	No (ref)	27,481	80.34	1
		<1 time	3360	9.82	2
		≥1 to <2 times	2653	7.76	3
		≥2 times	712	2.08	4

Notes: “ref” refers to “reference group”.

**Table 2:** Data distribution and assignment of continuous variables.

Variable	*N*	Min	Median	Max	Mean	Std
Level 1—individual						
Energy intake (kcal/day)	34,206	625.54	1825.29	4799.56	1912.48	591.92
Fat energy ratio (g/day)	34,206	2.55	34.42	84.30	34.82	11.86
Sodium intake (mg/day)	34,206	44.32	5491.16	11,999.89	5707.72	2750.88
Level 2—provincial						
Provincial GDP (100 million yuan)	31	1043.00	16,780.90	74,732.40	22,375.55	18,333.64
Catering industry turnover (100 million yuan)	31	0.50	96.70	670.00	156.91	183.12
Food consumption price index for food eaten out	31	101.10	102.50	105.80	102.64	1.12
Per capita daily consumption of edible oil (kg/day)	31	20.55	27.40	41.64	29.24	6.21
Per capita daily consumption of vegetable (kg/day)	31	67.67	254.79	364.11	258.11	55.85

Abbreviations: Min, Minimum value; Median, Median value; Max, maximum value; Mean, Mean value; Std, standard deviation.

**Table 3:** Fitting results of two-level logistic random intercept null model.

Parameter	Estimated Value	SE	t	*p*
$\beta_{0}$	−1.862	0.085	−21.83	<0.0001
$\sigma_{u_{0}}^{2}$	0.216	0.057	3.77	0.0007

Abbreviation: estimated value, β; SE, Standard error; t, t-test statistics of numerical integral approximation; *p*, hypothesis testing *p* Values.

**Table 4:** Fitting results of two-level multi-factor logistic model.

Variable	Exp (β) = OR	95% CI		t	*p*
Fixed effect					
Intercept	0.085	0.052	0.137	−10.53	<0.0001
Sex					
Male (ref)					
Female	0.955	0.893	1.023	−1.37	0.1798
Age, years					
18~44 (ref)					
45~59	1.165	1.088	1.249	4.54	<0.0001
Marital Level					
Spinsterhood (ref)					
Married/cohabitation	1.136	0.954	1.352	1.49	0.1458
Widowed/divorce/separation	1.102	0.823	1.476	0.68	0.4999
Employment					
Farming and aquaculture (ref)					
Others	1.104	1.013	1.203	2.35	0.0256
Student	1.419	0.825	2.443	1.32	0.1977
Unemployed/retired	1.249	1.144	1.365	5.14	<0.0001
Education Level					
Junior high and below (ref)					
High school/technical secondary school/technical school	0.897	0.819	0.982	−2.44	0.0207
Junior college and above	0.743	0.652	0.847	−4.63	<0.0001
Household Income Level, yuan					
<10,000 (ref)					
10,000~19,999	1.095	1.013	1.182	2.40	0.0230
≥20,000	1.013	0.927	1.108	0.30	0.7679
Frequency of Home, per day					
No (ref)					
<1 time	1.249	0.843	1.851	1.16	0.2562
≥1 to <2 times	1.263	0.876	1.821	1.31	0.2016
≥2 times	1.191	0.817	1.736	0.95	0.3501
Frequency of EADH, per day					
No (ref)					
<1 time	0.954	0.789	1.155	−0.50	0.6194
≥1 to <2 times	0.784	0.651	0.945	−2.67	0.0122
≥2 times	0.905	0.642	1.276	−0.59	0.5585
Frequency of EAFH, per day					
No (ref)					
<1 time	1.123	1.005	1.255	2.13	0.0419
≥1 to <2 times	1.249	1.091	1.431	3.35	0.0022
≥2 times	1.281	0.977	1.679	1.86	0.0721
Energy intake (kcal/day)	1.000	1.000	1.000	2.10	0.0447
Fat energy ratio (g/day)	1.002	0.999	1.005	1.43	0.1619
Sodium intake (mg/day)	1.000	1.000	1.000	0.41	0.6879
Provincial GDP (100 million yuan)	1.000	1.000	1.000	−0.08	0.9382
Catering industry turnover (100 million yuan)	1.000	0.999	1.002	0.45	0.6562
Food consumption price index for food eaten out	0.903	0.746	1.093	−1.09	0.2834
Per capita daily consumption of edible oil (kg/day)	1.000	0.970	1.030	−0.02	0.9821
Per capita daily consumption of vegetable (kg/day)	1.000	0.996	1.004	0.06	0.9537
Random effect					
Level 2					
Square deviation	1.245	1.104	1.403	3.73	0.0008

Notes: “ref” refers to “reference group"; Abbreviation: Exp (β) = OR, Odds Ratio; 95% CI, 95% confidence interval; t, t-test statistics of numerical integral approximation; *p*, hypothesis testing *p* Values. SE, Standard error.

**Table 5:** The fitting results of the optimized two-level multi-factor logistic prediction model.

Variable	Exp (β) = OR	95% CI		t	*p*
Fixed effect					
Intercept	0.091	0.059	0.140	−11.31	<0.0001
Age, years					
18~44 (ref)					
45~59	1.180	1.102	1.262	4.98	<0.0001
Employment					
Farming and aquaculture (ref)					
Others	1.112	1.020	1.211	2.52	0.0173
Student	1.291	0.764	2.184	0.99	0.3283
Unemployed/retired	1.236	1.133	1.348	5.00	<0.0001
Education Level					
Junior high and below (ref)					
High school/technical secondary school/technical school	0.900	0.823	0.986	−2.37	0.0244
Junior college and above	0.734	0.645	0.836	−4.85	<0.0001
Household Income Level, yuan					
<10,000 (ref)					
10,000~19,999	1.099	1.018	1.187	2.52	0.0172
≥20,000	1.017	0.930	1.111	0.38	0.7040
Frequency of Home, per day					
No (ref)					
<1 time	1.258	0.849	1.863	1.19	0.2421
≥1 or <2 times	1.277	0.886	1.840	1.37	0.1824
≥2 times	1.200	0.823	1.749	0.99	0.3305
Frequency of EADH, per day					
No (ref)					
<1 time	0.959	0.793	1.161	−0.44	0.6605
≥1 or <2 times	0.790	0.656	0.951	−2.59	0.0145
≥2 times	0.910	0.646	1.283	−0.56	0.5792
Frequency of EAFH, per day					
No (ref)					
<1 time	1.131	1.012	1.264	2.26	0.0313
≥1 or <2 times	1.258	1.099	1.440	3.47	0.0016
≥2 times	1.288	0.983	1.689	1.91	0.0655
Energy intake (kcal/day)	1.000	1.000	1.000	2.44	0.0207
Random effect					
Level 2					
Square deviation	1.254	1.110	1.418	3.79	0.0007

Notes: “ref” refers to “reference group"; Abbreviation: Exp (β) = OR, Odds Ratio; 95% CI, 95% confidence interval; t, t-test statistics of numerical integral approximation; *p*, hypothesis testing *p* Values. SE, Standard error.

## Data Availability

The data presented in this study are non-public.

## Appendix A

**Table A1:** Population distribution in 31 provinces.

Province	*n*	%
Beijing	827	2.42
Tianjin	597	1.75
Hebei	1336	3.91
Shanxi	824	2.41
Inner Mongolia	925	2.70
Liaoning	1238	3.62
Jilin	1010	2.95
Heilongjiang	1288	3.77
Shanghai	779	2.28
Jiangsu	1547	4.52
Zhejiang	1185	3.46
Anhui	1329	3.89
Fujian	1227	3.59
Jiangxi	1284	3.75
Shandong	2334	6.82
Henan	1436	4.20
Hubei	1189	3.48
Hunan	1458	4.26
Guangdong	1385	4.05
Guangxi	986	2.88
Hainan	881	2.58
Chongqing	593	1.73
Sichuan	1278	3.74
Guizhou	701	2.05
Yunnan	1346	3.93
Tibet	498	1.46
Shaanxi	1057	3.09
Gansu	1045	3.06
Qinghai	744	2.18
Ningxia	736	2.15
Xinjiang	1143	3.34

**Table A2:** Model fitting information.

Method	Model 1	Model 2	Model 3
−2LL	27,687	27,548	27,557
AIC	27,691	27,608	27,597
AICC	27,691	27,608	27,597
BIC	27,694	27,651	27,626
